# Comparative genome-based identification of a cell wall-anchored protein from *Lactobacillus plantarum* increases adhesion of *Lactococcus lactis* to human epithelial cells

**DOI:** 10.1038/srep14109

**Published:** 2015-09-15

**Authors:** Bo Zhang, Fanglei Zuo, Rui Yu, Zhu Zeng, Huiqin Ma, Shangwu Chen

**Affiliations:** 1 Key Laboratory of Functional Dairy, College of Food Science and Nutritional Engineering, China Agricultural University, Beijing, P. R. China; 2 College of Agriculture and Biotechnology, China Agricultural University, Beijing, China

## Abstract

Adhesion to host cells is considered important for *Lactobacillus plantarum* as well as other lactic acid bacteria (LAB) to persist in human gut and thus exert probiotic effects. Here, we sequenced the genome of *Lt. plantarum* strain NL42 originating from a traditional Chinese dairy product, performed comparative genomic analysis and characterized a novel adhesion factor. The genome of NL42 was highly divergent from its closest neighbors, especially in six large genomic regions. NL42 harbors a total of 42 genes encoding adhesion-associated proteins; among them, *cwaA* encodes a protein containing multiple domains, including five cell wall surface anchor repeat domains and an LPxTG-like cell wall anchor motif. Expression of *cwaA* in *Lactococcus lactis* significantly increased its autoaggregation and hydrophobicity, and conferred the new ability to adhere to human colonic epithelial HT-29 cells by targeting cellular surface proteins, and not carbohydrate moieties, for CwaA adhesion. In addition, the recombinant *Lc. lactis* inhibited adhesion of *Staphylococcus aureus* and *Escherichia coli* to HT-29 cells, mainly by exclusion. We conclude that CwaA is a novel adhesion factor in *Lt. plantarum* and a potential candidate for improving the adhesion ability of probiotics or other bacteria of interest.

*Lactobacillus plantarum* is a highly flexible and versatile species which can be found in various environmental as well as human intestinal niches^1^. This species is one of the food-grade lactic acid bacteria (LAB) that offers health-promoting properties to humans, including potential treatment effects for irritable bowel syndrome^2^ and recurrent *Clostridium difficile*-associated diarrhea^3^, protection of the epithelial barrier^4^, reduction of gastrointestinal symptoms during antibiotic treatment^5^, and cholesterol-lowering^6^ and immunomodulatory effects^7^,^8^. Specific strains of *Lt. plantarum*, 299v^8^ for example, are now being added to commercially available probiotic products^9^,^10^. In view of the beneficial health effects of *Lt. plantarum* to humans, much effort has been invested in isolating and screening new strains, which might have improved or new probiotic traits, from various environmental niches, including natural fermented foods, plants and the human body^11^,^12^,^13^.

Adhesion of probiotic bacteria to human intestinal epithelial cells may favor their persistence in the gut, allowing them to exert beneficial effects on the host^14^. Bacterial adhesion to host mucosa is often mediated by the interaction of cell-surface components, including receptor-specific binding and charge and hydrophobic interactions^15^; mucus and epithelial adhesion represent the early and late stages of adhesion, respectively^14^. Autoaggregation and hydrophobicity are two indirect methods of evaluating the adhesion ability of bacteria^16^,^17^. Different adhesion mechanisms and molecules have been revealed in *Lactobacillus*, including surface-layer (S-layer) proteins in *Lt. acidophilus*, *Lt. gasseri*, *Lt. johnsonii*, *Lt. crispatus* and *Lt. brevis*^15^, cell wall-anchored mucus-binding protein in *Lt. reuteri*^18^, cell-surface collagen-binding protein in *Lt. reuteri* NCIB11951^19^, mannose-specific adhesin in *Lt. plantarum*^20^, and the mucus-binding pilin SpaC in *Lt. rhamnosus* GG^21^, among others. In comparison, there is less information on *Lactococcus* adhesion because these bacteria are not traditionally considered to be natural colonizers of humans^22^. In recent years, however, the presence of proteins containing a mucus-binding domain^23^, pili encoded by plasmids^24^ and surface physicochemical properties of charge or hydrophobicity^25^ has been predicted or verified to be correlated with the adhesive properties of *Lc*. *lactis.*

Genetic manipulation is a potent approach to designing new probiotic strains with improved or novel probiotic traits^26^. Various bacterial or even human targets of interest, such as enzymes^27^, cytokines and/or antigens^28^,^29^, adhesion proteins^30^ and so forth have been verified to be functional in existing probiotics. As for adhesion, Koo *et al.*^30^ demonstrated that recombinant probiotic *Lt*. *paracasei* expressing *Listeria* adhesion protein effectively blocks adhesion, invasion, and translocation of *Listeria monocytogenes,* thereby aiding in the targeted clearance of *Listeria* infection. In addition, a newly identified *Bifidobacterium bifidum*-specific protein (BopA) involved in adhesion improved the adhesive properties of recombinant bifidobacteria^31^. Among the expression of different heterologous genes in LAB hosts, *Lc*. *lactis* has proven to be optimal for heterologous protein production and the delivery of therapeutic and prophylactic molecules^32^, mainly because *Lc. lactis* is considered to be a noninvasive and nonpathogenic organism which secretes relatively few proteins and does not produce extracellular proteases^33^.

We previously isolated 30 different LAB strains from traditional dairy products produced by herders in the western Tianshan Mountains of China. General features of these isolates, in particular their fermentative characteristics, were analyzed^34^. Among those isolates, a *Lt. plantarum* strain (NL42) displayed both high autolytic activity and high autoaggregation ability (reflecting potential high adhesive ability). To reveal the genome features of this isolate and to characterize its adhesion-associated factors, we sequenced the whole genome of *Lt. plantarum* NL42 and performed comparative genomic analysis. Based on this, a multidomain-containing, cell wall-anchored, adhesion-associated protein termed CwaA was predicted and features of *Lc*. *lactis* expressing this protein were characterized.

## Results

### Genome features and phylogeny of *Lactobacillus plantarum* strain NL42

**Figure 1:**
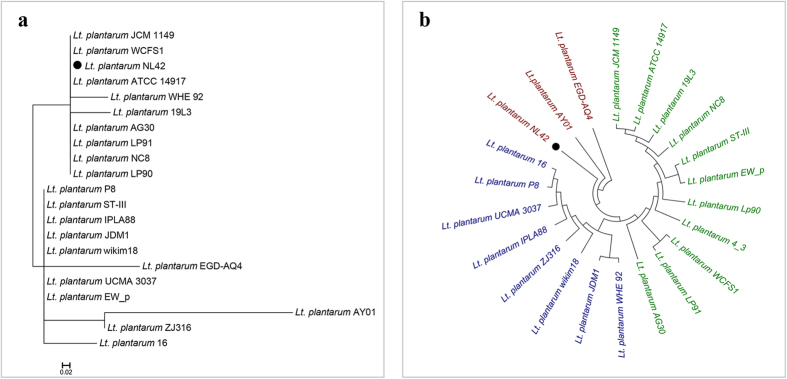
Phylogenetic analysis of different *Lt. plantarum* strains. (**a**) Genetic marker 16S rRNA gene-based and (**b**) whole-genome SNP-based phylogenetic trees. The trees were constructed using MEGA 5.1 software with the neighbor-joining method. The confidence of the trees was assessed by 1000-replicate bootstrapping. The scale bar in panel (**a**) means sequence divergence; while the tree shown in panel (**b**) is a topological structure in which three distinct clades are colored in red, blue and green, respectively. Only strains having complete or draft genome sequences in GenBank were included. The 16S rRNA gene of strain 4_3 was not available and thus is not presented in the marker gene-based tree.

The whole genome of *Lt*. *plantarum* NL42 was sequenced using the Illumina HiSeq 2000 platform. A total of 4,241,606 paired-end reads with a read length of 100 bp were generated, in total 848 M of raw data corresponding to 250-fold coverage of the genome. After quality filtering and assembly, we obtained the draft genome of NL42 consisting of 3,353,072 bp (52 contigs) with a GC content of 44.3% (Fig. S1). Rapid Annotation Using Subsystem Technology (RAST) annotation of the genome revealed 3,297 coding sequences (CDSs), 349 SEED subsystems, and 83 RNA genes. The 16S rRNA gene of NL42 was 100% identical to *Lt*. *plantarum* WCFS1, ATCC 14917, Lp90, LP91, AG30, NC8 and JCM 1149, and showed 99.6–99.9% similarity to the others (Fig. 1a and Fig. S2); however, whole-genome single nucleotide polymorphism (SNP)-based phylogenetic analysis grouped NL42 with AY01 and EGD-AQ4, forming a clade that was very distant from another two distinct clades. In addition, though NL42, AY01 and EGD-AQ4 were in the same clade, they were more divergent from each other than from members in the other two clades (Fig. 1b). These results suggested that even though its 16S rRNA genetic marker is closely related or even the same as those of other isolates, the NL42 genome is highly variable.

### Comparative genomics of *Lactobacillus plantarum*

**Figure 2:**
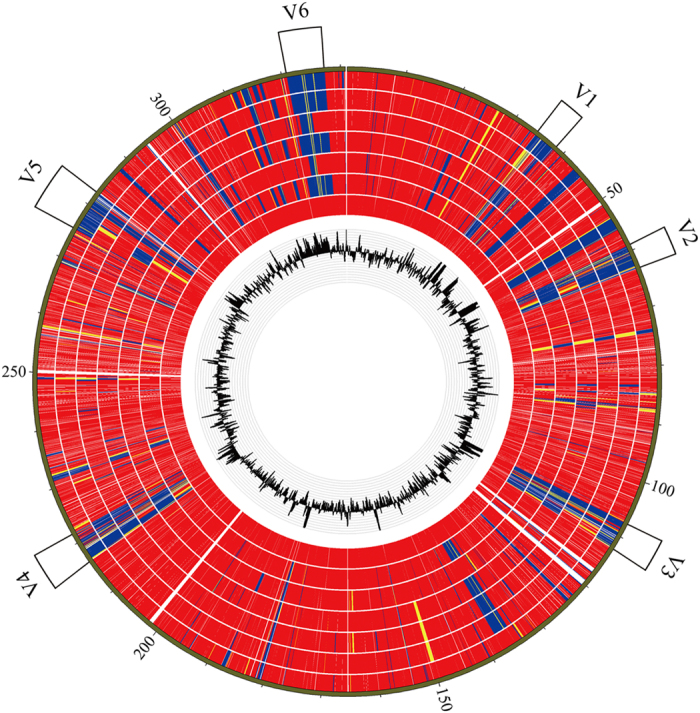
Comparison of protein sequence similarity among *Lt. plantarum* genomes. Protein sequences in NL42 and the other six complete genomes were aligned using WCFS1 as the reference genome. The innermost track shows the GC content of the reference. Rings from inside to outside are WCFS1, 16, JDM1, P8, ST_III, ZJ316 and NL42, respectively. Red, yellow and blue indicate 90–100%, 60–89% and less than 59% protein sequence identities, respectively. Six large and highly varied genomic regions (V1 to V6) are labeled outside the outer ring.

To reveal the genomic variations in NL42, we compared the CDSs of NL42 with six other available complete *Lt*. *plantarum* genomes, using WCFS1 as a reference. The results, shown in a heat map, revealed that the variations always occur in abnormal GC regions in the *Lt*. *plantarum* genomes (Fig. 2). Compared with WCFS1, NL42 displayed six large and highly varied genomic regions designated V1 to V6 (each covering more than 40 CDSs) (Data S1). According to their gene content, the functions of these six regions were predicted as follows: V1 (locus tags from lp_0373 to lp_0431 in WCFS1)—a bacteriocin biosynthesis gene cluster; V2 (lp_0624 to lp_0687)—prophage P1 locus; V3 (lp_1176 to lp_1233)—a polysaccharide biosynthesis gene cluster; V4 (lp_2399 to lp_2480)—prophage P2a and P2b loci; V5 (lp_3093 to lp_3164) and V6 (lp_3590 to lp_3650)—probably involved in sugar metabolism and transport, respectively. Notably, these variant regions were also present in the other *Lt*. *plantarum* genomes, and thus may be major contributors to the genome plasticity of this species.

We then compared the genes’ functional categories based on COG assignment among these *Lt*. *plantarum* genomes. The various *Lt. plantarum* genomes were found to harbor similar numbers of genes in each functional category (Fig. S3), with the highest number of genes assigned to the category ‘post-translational modification, protein turnover and chaperones’ (from 336 to 402 genes in the different genomes), followed by ‘cell wall/membrane/envelope biogenesis’ (270 to 305) and ‘replication, recombination and repair’ (251 to 299). Compared to the other six genomes, NL42 was slightly enriched in genes belonging to ‘transcription’ (263 genes), ‘lipid transport and metabolism’ (173 genes), ‘secondary metabolite biosynthesis, transport and catabolism’ (201 genes) and ‘cell motility’ (68 genes). We further sought and compared adhesion-associated proteins (adhesion-associated and cell wall anchor domain-containing proteins) in NL42 and the other six genomes. Strain ZJ316 harbored the highest number of these proteins (49 genes), whereas JDM1 had the lowest (40 genes) (Fig. S4). In general, proteins containing PepGly-associated (peptidoglycan-binding), cell wall anchor-associated and mucus-associated domains were the three most prevalent proteins in *Lt*. *plantarum*. Interestingly, NL42 was found to harbor a gene that encodes a protein containing five cell wall surface anchor repeat domains and an LPxTG-like cell wall anchor motif, termed cell wall-anchored protein A (CwaA). We then focused on characterizing this protein and its encoding gene, *cwaA*.

### CwaA is a multidomain-containing, cell wall-anchored, adhesion-associated protein

**Figure 3:**
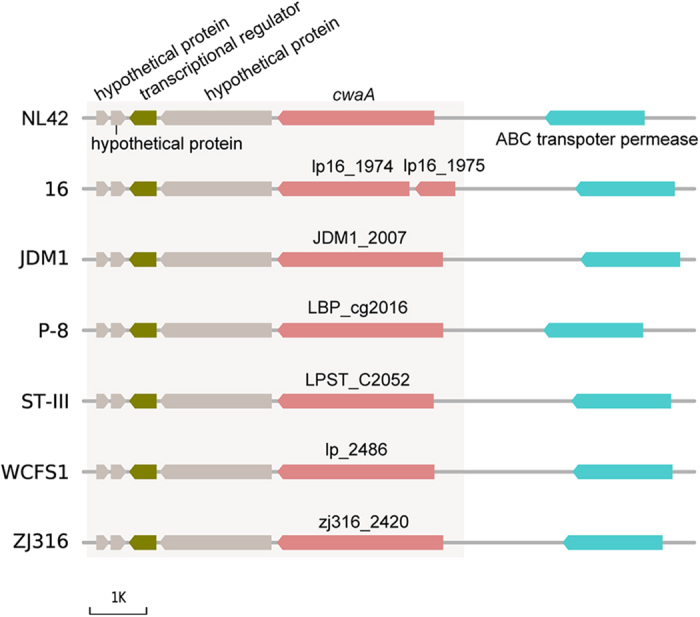
Genetic backgrounds of *cwaA* and its homologues in *Lt*. *plantarum* genomes. Gray-shaded regions indicate the putative operon composed of five different genes. A stop codon appears in the *cwaA* homologue in *Lt. plantarum* 16, resulting in the generation of two open reading frames, lp16_1974 and lp16_1975.

**Figure 4:**
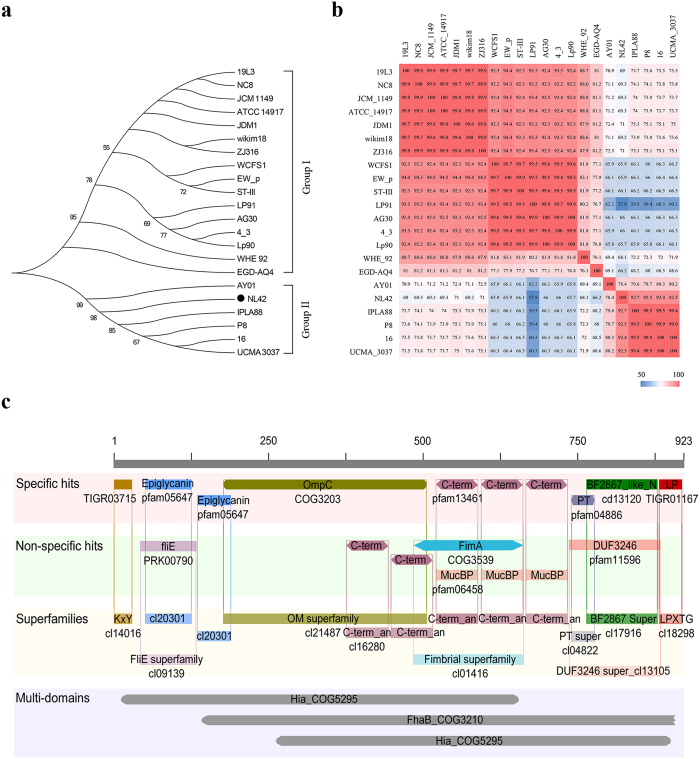
Phylogenetic relationship, nucleotide sequence diversity and conserved functional domains of CwaA. (**a**) Phylogenetic tree of *cwaA* gene and its homologues in 21 *Lt. plantarum* genomes. The tree was constructed using MEGA 5.1 software with the neighbor-joining method (1000-replicate bootstrapping). Bootstrap values are shown beside each node and the values less than 50% are not shown; (**b**) Heat-plot of the similarity matrices of *cwaA* gene in different genomes based on pairwise sequence alignments; and (**c**) Conserved functional domains in CwaA annotated by CDD. Different confidence levels are represented by specific hits and nonspecific hits, and the domain model scope includes superfamilies and multidomains. Specific hits indicate the top-ranking RPS-BLAST hits, meaning a high-confidence association between a query protein and a conserved domain; nonspecific hits meet or exceed the RPS-BLAST threshold for statistical significance; superfamilies are the domain clusters to which the specific and/or nonspecific hits belong; multidomains are domain models likely to contain multiple single domains.

The *cwaA* gene in the NL42 genome is probably the structural gene of an operon composed of five different open reading frames that encode three hypothetical proteins, a transcriptional regulator and the cell wall-anchored protein CwaA (Fig. 3). This putative operon structure was also found in the other six complete *Lt. plantarum* genomes. To further investigate the *cwaA* gene distribution and the sequence diversity, we searched and compared the homologues of *cwaA* in all 21 known *Lt. plantarum* genomes. Interestingly, *cwaA* homologues were found harbored by all the known *Lt. plantarum* genomes, with nucleotide identity ranging from 57.8% to 100% with *cwaA* in NL42. Phylogenetic analysis indicated that *cwaA* genes in the known *Lt. plantarum* genomes are clustered into two major groups, i.e., Group I and Group II. Most of the *Lt. plantarum* genomes (a total of 16) belong to Group I and only 6 genomes (NL42 included) are affiliated to Group II (Fig. 4a). The majority members among each group are similar to each other, showing more than 90% nucleotide identity, while members between the two groups are relative more divergent, usually less than 75% identity (Fig. 4b). Taken together, though the sequence of *cwaA* in different *Lt. plantarum* isolates are diverse and separately clustered, none of these genomes are devoid of *cwaA* homologues, suggesting that *cwaA* may play essential roles for this species.

The *cwaA* gene in NL42 is 2.772 kb long; it encodes 923 amino acids with a predicted molecular weight of 93.7 kD—47 strongly basic (+), 81 strongly acidic (−), 275 hydrophobic and 398 polar amino acids—with a secondary structure consisting mostly of β-sheets and turns (Fig. S5). The N terminus of CwaA is a KxYKxGKxW-type signal peptide (Fig. 4c), which tends to occur on long, low-complexity proteins of the phylum Firmicutes. The SignalP4.1 tool predicted a cleavage site between amino acid positions 48 and 49. The C terminus of CwaA contains an LPQTDE (LPxTG-like cell wall anchoring) motif belonging to the gram-positive LPxTG anchor superfamily. Interestingly, aside from the hexapeptide motif at the C terminus, CwaA possesses five cell wall surface anchor repeat domains (repeats 1 to 5, each 57 amino acids in length) (Fig. 4c and Fig. S6) which were first found in *L. monocytogenes*^35^. The LPxTG-like motif and three of the five cell wall surface anchor repeat domains (repeats 3 to 5) were all ranked as specific hit levels by the Conserved Domain Database (CDD) CD-Search tool, which represents a very high confidence level for the inferred function of the query protein^36^; we therefore concluded that CwaA is a cell wall-anchored protein. The specific hit domains of CwaA also included epiglycanin (tandem-repeating region of mucin, pfam05647), OmpC (outer membrane protein, COG3203), PT (the tetrapeptide XPTX repeat, pfam04886) and BF2867_like_N (N-terminal domain found in *Bacteroides fragilis* Nctc 9343 BF2867 and related proteins, cd13120), probably with a role in cell adhesion. Moreover, these specific hit domains also overlapped with other nonspecific hits; for example, the cell wall surface anchor repeats 3, 4 and 5 overlapped with MucBP (mucin binding protein) domains (pfam06458). Interestingly, when single domains were considered together (multidomain hit results), CwaA was more related to Hia (COG5295) and FhaB (COG3210) multidomains with e-values of 1.28e-20 and 1.30e-19, respectively. Hia and FhaB are, respectively, annotated as autotransporter adhesion and large exoproteins involved in heme utilization or adhesion. Taken together, these results strongly support CwaA as a multidomain-containing cell wall-anchored protein that is very likely involved in cell adhesion.

### Cell wall-anchored domains in CwaA are relatively conserved

Multiple sequence alignment of CwaA with its homologues in another five complete *Lt*. *plantarum* genomes indicated that the whole protein sequence of CwaA is most similar to hypothetical protein LBP_cg2016 in *Lt*. *plantarum* P8 (90.9% identity). However, the N-terminal signal peptide, the C-terminal LPxTG-like cell wall-anchoring motif and the cell wall surface anchor repeats 3, 4 and 5 of CwaA were nearly identical in these strains (Fig. S6). We further used the ConSurf server to analyze the conservation of amino acids in CwaA and all of its homologous sequences in the database. The results again showed that the amino acids in the regions mentioned above are more conserved (Fig. S7), suggesting that these amino acids per se and the cell wall-anchored domains containing them are critical to CwaA-like proteins.

### Overexpression of CwaA in *Lactococcus lactis*

**Figure 5:**
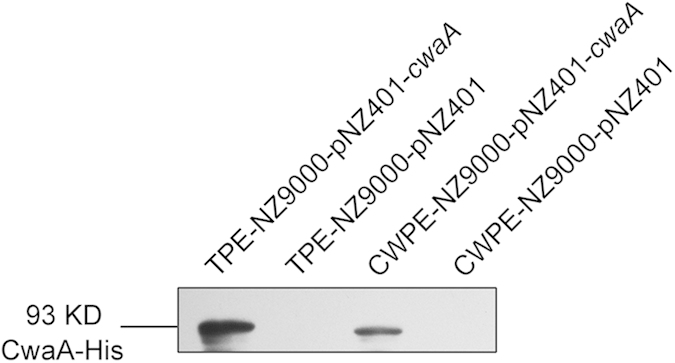
CwaA expressed in *Lc. lactis* detected by western blotting. TPE: total protein extract; CWPE: cell wall protein extract.

The *cwaA* gene in NL42 was cloned, 6×His-tagged and expressed in *L. lactis* NZ9000 using lactococcal expression vector pNZ401, resulting in the recombinant strain NZ9000-pNZ401-*cwaA*. Western blotting assay using an anti-His-tagged antibody revealed the expected 93-kD protein product in both the total protein extract and the cell wall-associated protein extract of this recombinant strain (Fig. 5). No corresponding products were found in the parent strain (NZ9000) harboring the empty vector. These results indicated that *cwaA* is efficiently expressed in *Lc. lactis* and the presence of its expression product CwaA in the cell wall protein extracts further proved that the protein is anchored to the cell wall.

### CwaA increases adhesion of *Lactococcus lactis* to HT-29 cells

**Figure 6:**
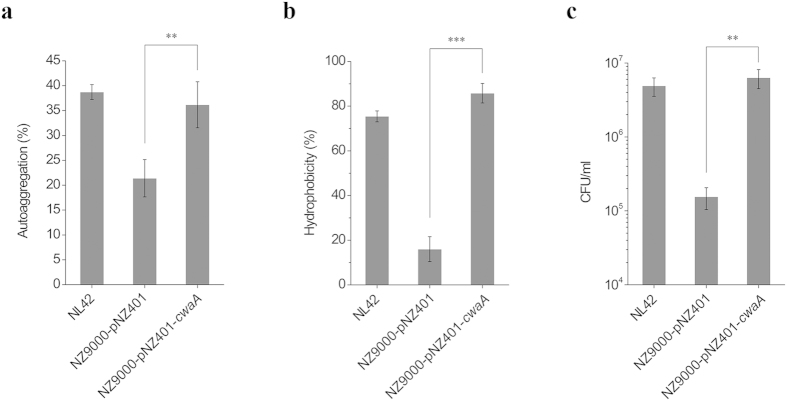
(**a**) Autoaggregation, (**b**) hydrophobicity and (**c**) adhesion properties of *Lc*. *lactis* expressing CwaA. Data are presented as means ± SEM of three independent experiments. *P ≤ 0.05, **P ≤ 0.01, ***P ≤ 0.001.

To evaluate whether CwaA is involved in adhesion, we first performed autoaggregation and hydrophobicity assays. Compared with the negative control strain NZ9000-pNZ401, the autoaggregation and hydrophobicity rates of NZ9000-pNZ401-*cwaA* were 1.8-fold and 5.4-fold higher, respectively (Fig. 6a,b), reaching 33.9% and 85.8%, which was comparable to the levels of *Lt*. *plantarum* NL42 (38.7% and 75.4%, respectively). Interestingly, CwaA seemed to be more proficient at improving *Lc. lactis* hydrophobicity (*P* < 0.001); the hydrophobicity rate of NZ9000-pNZ401-*cwaA* was even higher than that of NL42 (Fig. 6b). We then performed adhesion assays using the human colonic epithelial cell line HT-29 as a model. Similar to the trends in the autoaggregation and hydrophobicity assays, CwaA significantly improved the adhesive ability of *Lc. lactis* NZ9000, with 40-fold increase in the number of adherent bacterial cells (*P* < 0.01), representing a binding efficiency approaching that of NL42 (Fig. 6c). Taken together, these results confirmed that autoaggregation and hydrophobicity of *Lc. lactis* are closely correlated with its adhesive ability; CwaA played a critical role in autoaggregation and hydrophobicity improvement and thus increased the adhesion of *Lc. lactis* to HT-29 cells.

### *Lactococcus lactis* expressing CwaA blocks adhesion of pathogens

**Figure 7:**
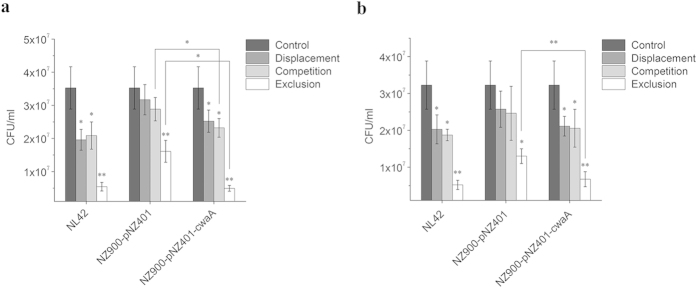
Inhibition effects of *Lc. lactis* expressing CwaA on the adhesion of (a) *S. aureus* and (b) enterotoxigenic *E. coli* (ETEC). Adhesion of pathogen to HT-29 cells without added *Lc. lactis* or *Lt*. *plantarum* served as the control. Displacement, competition and exclusion indicate that *Lc*. *lactis* or *Lt. plantarum* was incubated before, simultaneously with and after the pathogens, respectively. Data are presented as means ± SEM of three independent repeats. *P ≤ 0.05, **P ≤ 0.01.

To determine whether the increased adhesion of *Lc. lactis* inhibits adhesion of pathogenic bacteria and to elucidate the mode of action, we used *Staphylococcus aureus* and enterotoxigenic *Escherichia coli* (ETEC) as indicators in displacement, competition and exclusion blockage assays, performed by incubating *Lc*. *lactis* or *Lt*. *plantarum* before (displacement), simultaneously with (competition) or after (exclusion) the pathogens. In general, *Lt*. *plantarum* and *Lc*. *lactis* both inhibited the adhesion of the two pathogens to HT-29 cells; the greatest blockage effects were observed under conditions of exclusion. Compared with *Lc*. *lactis* NZ9000-pNZ401, recombinant strain NZ9000-pNZ401-*cwaA* further reduced the number of both pathogens adhered to HT-29 cells by approximately 40%, 20% and 20% under conditions of displacement, competition and exclusion, respectively. In the case of *S. aureus*, NZ9000-pNZ401-*cwaA* significantly reduced the number of adherent cells under conditions of competition and exclusion compared with NZ9000-pNZ401 (*P* < 0.05) (Fig. 7a), whereas for ETEC, this trend was only observed under condition of exclusion (Fig. 7b). The results suggested that the improved adhesion of *Lc*. *lactis* enhances the blocking effects on *S. aureus* and ETEC adhesion to HT-29 cells, and that exclusion, i.e. occupation of the adhesion sites by *Lc. lactis* expressing CwaA prior to the pathogens, was the most effective blocking mode.

### Cell-surface protein serves as a major receptor for CwaA adherence

**Figure 8:**
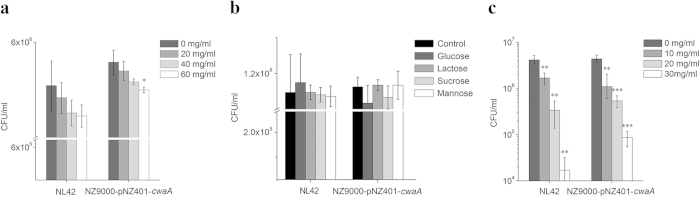
Adhesion of *Lc. lactis* expressing CwaA after (a) periodate treatment, (b) sugar inhibition and (c) protease treatment. Data are presented as means ± SEM of three independent experiments. *P ≤ 0.05, **P ≤ 0.01, ***P ≤ 0.001.

To reveal which surface components of the HT-29 cell are targets for CwaA adhesion, we treated the cells prior to NZ9000-pNZ401-*cwaA* adhesion with periodate or protease (trypsin) to investigate the contributions of carbohydrate and protein factors, respectively. Periodate treatment of HT-29 cells seemed to have a concentration-dependent but not statistically significant effect on both *Lt*. *plantarum* NL42 and *Lc*. *lactis* NZ9000-pNZ401-*cwaA* adhesion (Fig. 8a). Significantly reduced adhesion to HT-29 cells was only observed for NZ9000-pNZ401-*cwaA* under treatment with a high periodate concentration (60 mg/ml) (*P* < 0.05). These results suggested that the surface carbohydrate moieties of HT-29 cells are probably not the major receptor for CwaA adhesion. This was further supported by the results of sugar-inhibition tests in which glucose, lactose, sucrose and mannose had no obvious competitive inhibitory effects on either NL42 or NZ9000-pNZ401-*cwaA* adhesion (*P* > 0.05 for each) (Fig. 8b). In contrast, trypsin treatment of HT-29 cells significantly reduced adhesion of both NL42 and NZ9000-pNZ401-*cwaA*, and a significant reduction was even observed at the low trypsin concentration of 10 mg/ml (*P* < 0.01 and 0.001 for NL42 and NZ9000-pNZ401-*cwaA*, respectively) (Fig. 8c). In addition, bacterial cell binding to the HT-29 cells decreased gradually with increasing trypsin concentration, showing a strong concentration-dependent trend. Taken together, we suggest that a certain kind of protein, but not carbohydrate moieties, on the HT-29 cell surface is the major receptor for CwaA adhesion.

## Discussion

*Lt*. *plantarum* is considered a flexible and versatile LAB^1^. This is reflected, to some extent, by the analysis provided herein, showing that although the 16S rRNA gene of strain NL42 is the same as that of strain WCFS1 and others, the genomes are highly divergent. The NL42 genome was similar in size to that of WCFS1 (the first *Lt. plantarum* genome), but was highly varied in six large genomic abnormal GC regions; two of these were prophage loci, suggesting the large contribution of horizontal gene transfer mediated by mobile genetic elements to the genome plasticity of this species. In addition, the variations in gene clusters related to polysaccharide biosynthesis (V3) and sugar metabolism and transport (V6), and the gene enrichment in transcription and lipid transport and metabolism might reflect NL42’s adaption to or fitness in the dairy environment.

A mannose-specific adhesion mechanism has been reported in *Lt. plantarum* strains 299 and 299v, and a mannose-specific adhesin (Msa) has been identified in WCFS1 (gene locus, lp_1229)^20^. Here we found that lp_1229 is located in the V3 region and is lost in strain NL42 (Data S1), suggesting that adhesion of NL42 does not occur in a mannose-specific manner and that Msa is not the major adhesin in this strain. This assumption is also supported by our sugar-inhibition test results in which mannose did not inhibit the adhesion of NL42.

Cell wall-anchored surface proteins, especially those with an N-terminal LPxTG-like motif, have frequently been identified to be involved in adhesion in LAB as well as bacterial pathogens^37^,^38^. CwaA was identified as a cell wall-anchored adhesion-associated protein based on the facts that: 1) it contains an LPxTG-like motif, 2) it harbors five cell wall surface anchor repeat domains, three of them overlapping with MucBP domains, 3) its multidomain is similar to those of other adhesion-associated proteins, and most importantly 4) it enhances autoaggregation and hydrophobicity of *Lc*. *lactis*, thereby providing it with the ability to adhere. CwaA was similar (90.9% amino acid identity) to a hypothetical protein of another isolate originating from a traditional Chinese dairy product—*Lt*. *plantarum* P8^39^—and showed 67.3% identity with WCFS1 lp_2486 which has been annotated as a “mucus-binding protein, LPxTG-motif cell wall anchor”, probably due to overlap of the putative MucBP domains with the cell wall surface anchor repeat domains in CwaA. However, our CCD CD-Search results suggested that the cell wall surface anchor repeat domains are ranked as specific hits in the database, i.e., top-ranking RPS-BLAST hits compared to other hits in overlapping intervals, whereas the MucBP domains were ranked as nonspecific hits. We therefore named this protein cell wall-anchored protein A to reflect its features according to the specific domains it contains.

The LPxTG-like motif-containing proteins are sorted and covalently coupled to the cell wall by sortase in gram-positive bacteria^40^. A previous study indicated that sortase A (SrtA) of *Lc*. *lactis* has different LPxTG-like motif-containing substrates, such as LPKTGE, LPFTGG, LPETGD and LPSTGD^41^. The successful expression of CwaA (LPQTDE) in *Lc. lactis* prompts us to suggest that LPQTxE, an LPxTG-like sorting motif in cell wall-bound proteins of *Lt*. *plantarum*^42^, is another potential substrate of *Lc*. *lactis* sortase. Actually, *Lt*. *plantarum* NL42 SrtA and *Lc*. *lactis* NZ9000 SrtA shared more than 60% amino acid similarity (data not shown).

Different adhesion-associated proteins of either probiotic or pathogen origin, including the collagen-binding S-layer protein CbsA of *Lt*. *crispatus*^43^, the N-terminal region of the S-layer protein SlpA of *Lt. brevis*^44^, cell wall-associated polypeptides SspA and SspB of *Streptococcus gordonii*^45^, the lipoprotein BopA of *Bifidobacterium bifidum*^31^, and the pneumococcal surface protein PspC of *Streptococcus pneumoniae*^46^ have been characterized and demonstrated to confer adhesive properties to *Lc*. *lactis* or others with varying degrees. For examples, *Lc. lactis* MG1363 expressing cell surface *Streptococcus gordonii* SspA and SspB exhibited 10-fold- and 5-fold-increased binding, respectively^45^, to immobilized salivary agglutinin glycoprotein compared with controls; and the adhesion abilities of *B. longum/infantis* E18 to T84, Caco-2 and HT-29 cells were improved by 511%, 180% and 209%, respectively^32^. Here, CwaA increased the number of *Lc. lactis* NZ9000 adhering to HT-29 cells by about 40-fold, leading to the recombinant strain nearly reaches the level of the adhesive ability of CwaA’s original host, *Lt. plantarum* NL42, suggesting that it is a favorable candidate for improving the adhesion of *Lc. lactis*. However, compared with the degree of the improvement of adhesion, the pathogen blocking effects of *Lc. lactis* expressing CwaA is relative minor (Fig. 6C). These results prompt us to suggest that the adhesion sites of CwaA and the pathogens tested are not completely overlapped. For example, the *E. coli* ETEC H10407 we used has been demonstrated to use different strategies to adhere to human epithelial cells, such as using adhesins Tia and Tib and through the interaction of exoprotein EtpA and flagella^47^,^48^.

In summary, we sequenced the whole genome of a *Lt*. *plantarum* strain NL42 originally isolated from a traditional Chinese dairy product. Comparative genomic analysis of this genome with other available *Lt. plantarum* genomes was performed, predicting a candidate cell wall-anchored adhesion-associated protein, CwaA, in *Lt. plantarum*. CwaA conferred *Lc*. *lactis* strain NZ9000 with significantly improved adhesive ability to HT-29 cells, probably via adhesion to a surface protein on these cells. In addition, The *Lc. lactis* expressing CwaA not only acquired improved adhesion capability, but also blocked the adhesion of bacterial pathogens. We therefore expect that a recombinant *Lc. lactis* with these properties would be most valuable for future efficient delivery of interesting molecules. Furthermore, we should stress that future efforts are still needed to address the issues including the specific target of CwaA and the attachment mechanism, the core functional domains of CwaA, the relationship between *cawA* gene diversity and its adhesive characteristics, and the efficiency of CwaA to improve the probiotic traits *in vivo*.

## Methods

### Bacterial strains and growth conditions

The strains and plasmids used in this study are listed in Table S1. *Lt*. *plantarum* strain NL42 was grown anaerobically at 37 °C in MRS broth (Difco Laboratories, Detroit, MI) for 16 h. *Lactococcus* strains were cultured in M17 (Difco) containing 0.5% (w/v) glucose at 30 °C without shaking. When required, erythromycin was added at 5 μg/ml. The *S. aureus* and *E. coli* were incubated in LB medium containing 1% (w/v) tryptone, 0.5% (w/v) yeast extract and 1% (w/v) NaCl at 37 °C with shaking at 220 rpm for 16 h.

### Genome sequencing and comparative genomics

The whole genome of *Lt*. *plantarum* NL42 was sequenced on an Illumina HiSeq 2000 platform according to a standard protocol. Genome assembly and annotation were performed with SOAPdenovo (http://soap.genomics.org.cn) and RAST programs (Rapid Annotation using Subsystem Technology)^49^, respectively. Available *Lt*. *plantarum* genomes (6 complete and 15 draft sequences) were retrieved from NCBI GenBank and whole-genome alignment and SNP calling were performed using Mugsy^50^. Protein sequence similarity among genomes was determined by BLASP against the reference genome WCFS1 and visualized as a circle heat map using Circos^51^. Multiple sequence alignment of CwaA with its homologues in other genomes was performed by Clustal X version 2.0^52^. Gene functional categories were analyzed using the COG database^53^. Adhesion- and cell wall anchor-associated domain-containing proteins were searched by BLAST against the Pfam protein families database^54^.

### Other bioinformatics tools

MEGA software (version 5)^55^ was used to construct the phylogenetic trees. The CD-Search tool in the CDD (Conserved Domain Database)^36^ was used to search for conserved domains and functional annotations in CwaA. SignalP4.1 server (http://www.cbs.dtu.dk/services/SignalP/) was used to predict the CwaA signal peptide sequence and cleavage site. The evolutionary conservation of amino acids in CwaA was estimated with the help of ConSurf server (http://consurf.tau.ac.il/). The secondary structure of CwaA was predicted using Protean (Lasergene package, DNASTAR, Madison, WI).

### Cloning and inducible expression

The *cwaA* gene was amplified from *Lt*. *plantarum* NL42 chromosomal DNA using primers of cwaAF (5′-GCTCTAGAATGTCAAAAGATAATCAAAAA-3′, *Xba*I site underlined) and cwaAR (5′-CCGCTCGAGTTA*GTGGTGGTGGTGGTGGTG*TGCTTCATGCTTCCGACGAGA-3′, *Xho*I site underlined), and sequence coding for a 6 × His tag (italics) was incorporated into the reverse primer. The PCR product was then cloned into plasmid pNZ401 between *Xba*I and *Xho*I sites, resulting pNZ401-*cwaA*. Plasmids pNZ401-*cwaA* and empty control pNZ401 extracted from *E. coli* DH5α were both transformed into *Lc*. *lactis* NZ9000. For inducible expression, the recombinant *Lc*. *lactis* NZ9000-401-*cwaA* and control (NZ9000-401) were grown overnight in glucose-M17 (GM17) medium containing 5 μg/ml erythromycin. A 2% (v/v) inoculum was transferred to fresh GM17 broth and grown at 30 °C without shaking to an optical density at 600 nm (OD_600_) of 0.4 to 0.6, and then 10 ng/ml nisin was added and the culture was incubated for 3 h before harvesting.

### Western blot analysis

The induced recombinant *Lc*. *lactis* was harvested by centrifugation at 8000 *g* for 10 min at 4 °C. Pelleted cells were washed three times in PBS, resuspended in PBS and disrupted by ultrasonicator. The supernatant was mixed with 5 × SDS loading buffer (250 mM Tris-HCl pH 6.8, 10% w/v SDS, 0.5% w/v bromophenol blue, 50% v/v glycerol, 5% w/v β-mercaptoethanol) and separated by 12% SDS–PAGE, and then transferred to a nitrocellulose membrane using a semi-dry transmembrane system. Protein bands were detected using a His-Tag XP^@^ Rabbit monoclonal antibody. The *L*. *lactis* NZ9000 harboring empty vector pNZ401 was used as a control. Cell wall proteins were extracted as described previously^56^.

### Autoaggregation and hydrophobicity assays

Autoaggregation assays were carried out according to Collado *et al.*^57^ with some modifications. The bacterial cells were harvested by centrifugation at 8000 *g* for 10 min, washed twice in PBS and resuspended in PBS to an OD_600_ of around 0.5. The bacterial suspensions were then mixed by vortexing and incubated at 30 °C for 4 h. Autoaggregation percentage was calculated using the formula: 1−(A_4_/A_0_) × 100%, where A_4_ represents OD_600_ at 4 h and A_0_ the absorbance at 0 h. In the hydrophobicity assay, 1 ml xylene was added to 3 ml cell suspension; the mixture was shaken by vortexing for 90 s and incubated at room temperature for 20 min, and then the OD_600_ of the aqueous phase was measured. The percentage of hydrophobicity was expressed as [(A_0_−A)/A_0_] × 100%, where A_0_ and A are the absorbance before and after extraction with xylene, respectively.

### HT-29 cell culture and adhesion assay

Human colonic epithelial HT-29 cells were cultured in Dulbecco’s Modified Eagle Medium (DMEM) containing 10% (v/v) fetal calf serum, and 100 U/ml of penicillin/streptomycin. For adhesion assays, 10^5^ HT-29 cells were seeded in 24-well plates with glass cover slips, and maintained at 37 °C under 5% CO_2_ for 3 days. Prior to the experiments, all bacterial cultures were harvested until the stationary phase or after induction and washed twice in PBS. Bacterial cells of 10^8^ or 10^9^ CFU/ml dissolved in 1 ml DMEM were inoculated into each well containing HT-29 cells. After co-incubation for 4 h at 37 °C, 5% CO_2_, the HT-29 cells were washed five times in PBS to remove the free bacterial cells and then lysed with 1 ml Triton X-100 (1% v/v) in PBS. The cell lysates were serially diluted and plated on agar plates.

### Treatment of cells with sodium periodate and trypsin

Washed HT-29 cells were disposed with different concentrations of sodium periodate or trypsin (37 °C, 30 min) as previously described^58^, and then adhesion experiments were performed. Acetate buffer (0.2 M, pH 4.6) was used to dilute sodium periodate and trypsin, and alone as a control. The sodium periodate was used at 0, 20, 40 and 60 mg/ml, and the trypsin was used at 0, 10, 20 and 30 mg/ml.

### Sugar-inhibition tests

Four types of sugar—glucose, mannose, sucrose and lactose—were tested for their ability to competitively inhibit adhesion. The adhesion assay was carried out in the presence of a sugar at 20 mg/ml, and no sugar was added to the corresponding control.

### Blocking pathogen adhesion

To investigate the ability of the recombinant *Lc*. *lactis* to block the adhesion of pathogens to HT-29 cells, *S. aureus* ATCC 25923 and *E. coli* ETEC H10407 (ATCC 35401) were used for displacement, competition and exclusion assays as described previously^59^. The *S. aureus* ATCC 25923 is a widely used indicator for both antimicrobial activity and adhesion assays, and the *E. coli* ETEC H10407 (serotype O78:H11) is an enterotoxin producer, which was originally isolated from human feces^60^. Briefly, in the displacement assay, 500 μl pathogens (10^8^ CFU/ml) and HT-29 cells (10^6^) were incubated together (37 °C, 2 h) and then 500 μl *Lc. lactis* (10^8^ CFU/ml) was added later and incubated for another 2 h; in the competition assay, *Lc. lactis*, pathogens and HT-29 cells were incubated together (37 °C, 4 h); in the exclusion assay, *Lc. lactis* and HT-29 cells were incubated together (37 °C, 2 h) and then 500 μl pathogens was added and the mixture incubated for another 2 h. After the adhesion incubation, HT-29 cells were washed five times with PBS and lysed with 1 ml Triton X-100 (1% in PBS). The cell lysates were serially diluted and plated on a LB agar plate.

### Statistics

Statistical analyses were performed using unpaired two-tailed Student’s t-tests. *P* values less than 0.05 were considered to be statistically significant. Data are presented as means ± SEM of three independent repeats in each experiment.

### Nucleotide sequence accession numbers

The genome sequence of *Lt*. *plantarum* NL42 and the nucleotide sequence of the *cwaA* gene have been deposited in the GenBank database under accession numbersJZSB00000000 and KP893285, respectively.

## Additional Information

**How to cite this article**: Zhang, B. *et al.* Comparative genome-based identification of a cell wall-anchored protein from *Lactobacillus plantarum* increases adhesion of *Lactococcus lactis* to human epithelial cells. *Sci. Rep.* **5**, 14109; doi: 10.1038/srep14109 (2015).

## Supplementary Material

Supplementary Information

Supplementary Dataset 1
