# Supplementary material for: Comparative genome-based identification of a cell wall-anchored protein from Lactobacillus plantarum increases adhesion of Lactococcus lactis to human epithelial cells
Source: Sci Rep. 2015 Sep 15;5:14109. doi: 10.1038/srep14109 (PMC4572922; doi:10.1038/srep14109)
Supplement: Supplementary Information [file srep14109-s1.pdf]

Supplementary Information

Comparative genome-based identification of a cell wall-anchored protein from *Lactobacillus plantarum* increases adhesion of *Lactococcus lactis* to human epithelial cells

Bo Zhang[1], Fanglei Zuo[1], Rui Yu[1], Zhu Zeng[1], Huiqin Ma[2], Shangwu Chen[1]*

[1] *Key Laboratory of Functional Dairy, College of Food Science and Nutritional Engineering, China Agricultural University, Beijing, P. R. China*

[2]*College of Agriculture and Biotechnology, China Agricultural University, Beijing, China*

*Corresponding author. Mailing address for Shangwu Chen: College of Food Science and Nutritional Engineering, China Agricultural University, No. 17 Qinghua East Road, Hai-dian District, Beijing 100083, China. Phone: (86)1062738663. Fax: (86)1062738663. E-mail: swchen@cau.edu.cn.

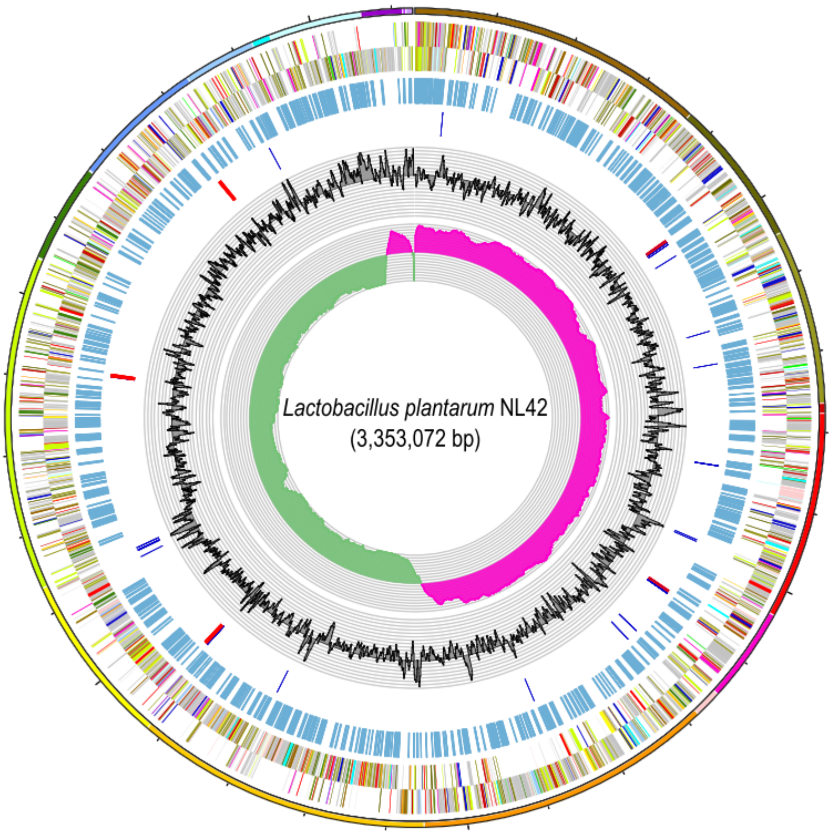

Fig. S1 Genome map of *Lt*. *plantarum* NL42. The circles from inside to outside indicate: GC skew (ring 1); GC content (ring 2); RNA genes (ring 3); KEGG-annotated enzymes (ring 4); COG-annotated coding sequences (rings 5 and 6) and genome sequence (ring 7, each tick indicates 100 kb). The order of the contigs was arranged according to *Lt*. *plantarum* WCFS1.

Percent Identity (upper right); Divergence (lower left)

	1	2	3	4	5	6	7	8	9	10	11	12	13	14	15	16	17	18	19	20	21		
1		99.9	99.9	99.9	**99.9**	100.0	100.0	100.0	99.9	99.5	100.0	99.9	99.9	99.9	99.9	99.9	99.5	99.9	100.0	100.0	99.9	1	JDM1
2	0.1		100.0	100.0	**100.0**	99.9	99.9	99.9	100.0	99.6	99.9	99.9	99.9	99.9	100.0	100.0	99.4	99.8	99.9	99.9	100.0	2	Lp90
3	0.1	0.0		100.0	**100.0**	99.9	99.9	99.9	100.0	99.6	99.9	99.9	99.9	99.9	100.0	100.0	99.4	99.8	99.9	99.9	100.0	3	LP91
4	0.1	0.0	0.0		**100.0**	99.9	99.9	99.9	100.0	99.6	99.9	99.9	99.9	99.9	100.0	100.0	99.4	99.8	99.9	99.9	100.0	4	NC8
**5**	**0.1**	**0.0**	**0.0**	**0.0**		**99.9**	**99.9**	**99.9**	**100.0**	**99.6**	**99.9**	**99.9**	**99.9**	**99.9**	**100.0**	**100.0**	**99.4**	**99.8**	**99.9**	**99.9**	**100.0**	**5**	**NL42**
6	0.0	0.1	0.1	0.1	**0.1**		100.0	100.0	99.9	99.5	100.0	99.9	99.9	99.9	99.9	99.9	99.5	99.9	100.0	100.0	99.9	6	P8
7	0.0	0.1	0.1	0.1	**0.1**	0.0		100.0	99.9	99.5	100.0	99.9	99.9	99.9	99.9	99.9	99.5	99.9	100.0	100.0	99.9	7	ST-III
8	0.0	0.1	0.1	0.1	**0.1**	0.0	0.0		99.9	99.5	100.0	99.9	99.9	99.9	99.9	99.9	99.5	99.9	100.0	100.0	99.9	8	UCMA_3037
9	0.1	0.0	0.0	0.0	**0.0**	0.1	0.1	0.1		99.6	99.9	99.9	99.9	99.9	100.0	100.0	99.4	99.8	99.9	99.9	100.0	9	WCFS1
10	0.1	0.1	0.1	0.1	**0.1**	0.1	0.1	0.1	0.1		99.5	99.5	99.5	99.5	99.6	99.5	98.9	99.4	99.5	99.5	99.6	10	WHE_92
11	0.0	0.1	0.1	0.1	**0.1**	0.0	0.0	0.0	0.1	0.1		99.9	99.9	99.9	99.9	99.9	99.5	99.9	100.0	100.0	99.9	11	wikim18
12	0.1	0.1	0.1	0.1	**0.1**	0.1	0.1	0.1	0.1	0.2	0.1		99.9	99.8	99.9	99.9	99.5	99.8	99.9	99.9	99.9	12	ZJ316
13	0.1	0.1	0.1	0.1	**0.1**	0.1	0.1	0.1	0.1	0.2	0.1	0.1		99.8	99.9	99.9	99.4	99.8	99.9	99.9	99.9	13	16
14	0.1	0.1	0.1	0.1	**0.1**	0.1	0.1	0.1	0.1	0.1	0.1	0.2	0.2		99.9	99.9	99.4	99.7	99.9	99.9	99.9	14	19L3
15	0.1	0.0	0.0	0.0	**0.0**	0.1	0.1	0.1	0.0	0.1	0.1	0.1	0.1	0.1		100.0	99.4	99.8	99.9	99.9	100.0	15	AG30
16	0.1	0.0	0.0	0.0	**0.0**	0.1	0.1	0.1	0.0	0.1	0.1	0.1	0.1	0.1	0.0		98.5	99.8	99.9	99.9	100.0	16	ATCC_14917
17	0.2	0.3	0.3	0.3	**0.3**	0.2	0.2	0.2	0.3	0.3	0.2	0.1	0.3	0.3	0.3	0.6		99.4	99.5	99.5	99.4	17	AY01
18	0.1	0.2	0.2	0.2	**0.2**	0.1	0.1	0.1	0.2	0.3	0.1	0.2	0.2	0.3	0.2	0.2	0.3		99.9	99.9	99.8	18	EGD-AQ4
19	0.0	0.1	0.1	0.1	**0.1**	0.0	0.0	0.0	0.1	0.1	0.0	0.1	0.1	0.1	0.1	0.1	0.2	0.1		100.0	99.9	19	EW_p
20	0.0	0.1	0.1	0.1	**0.1**	0.0	0.0	0.0	0.1	0.1	0.0	0.1	0.1	0.1	0.1	0.1	0.2	0.1	0.0		99.9	20	IPLA88
21	0.1	0.0	0.0	0.0	**0.0**	0.1	0.1	0.1	0.0	0.1	0.1	0.1	0.1	0.1	0.0	0.0	0.3	0.2	0.1	0.1		21	JCM_1149
	1	2	3	4	5	6	7	8	9	10	11	12	13	14	15	16	17	18	19	20	21		

Fig. S2 Sequence distance of the 16S rRNA genes of *Lt. plantarum*. Strains used here are the same with those in Fig 1a. Strain names represented by the numbers 1 to 21 are listed on the right.

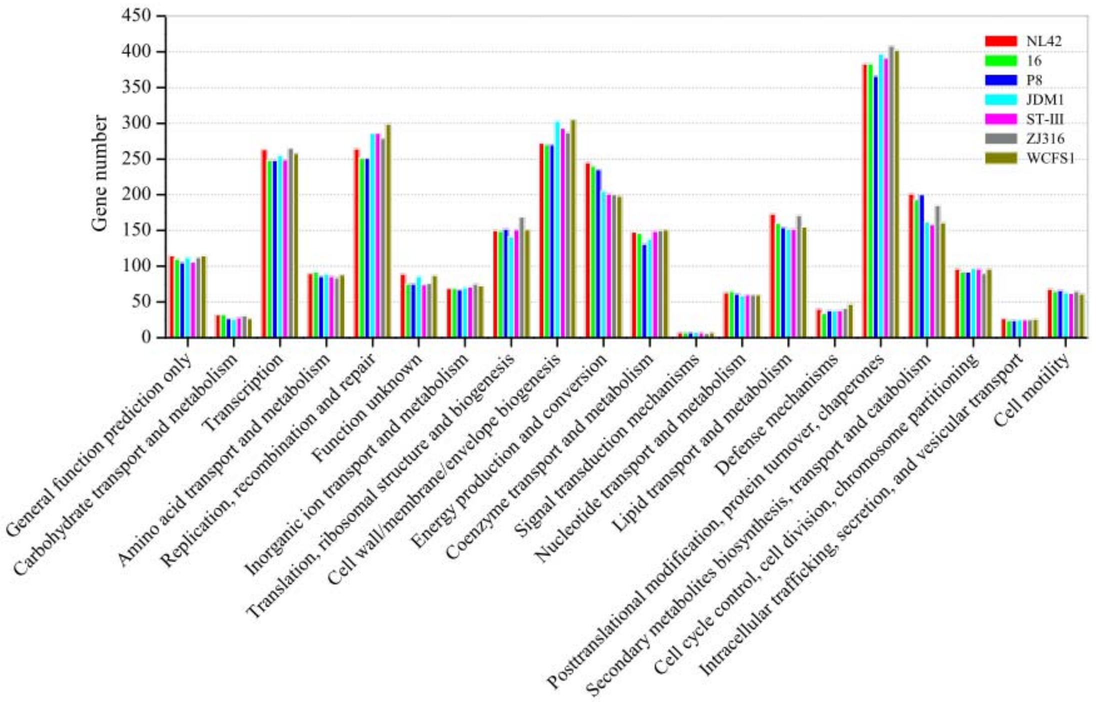

Fig. S3 Numbers of genes assigned to different COG functional categories among *Lt. plantarum* genomes.

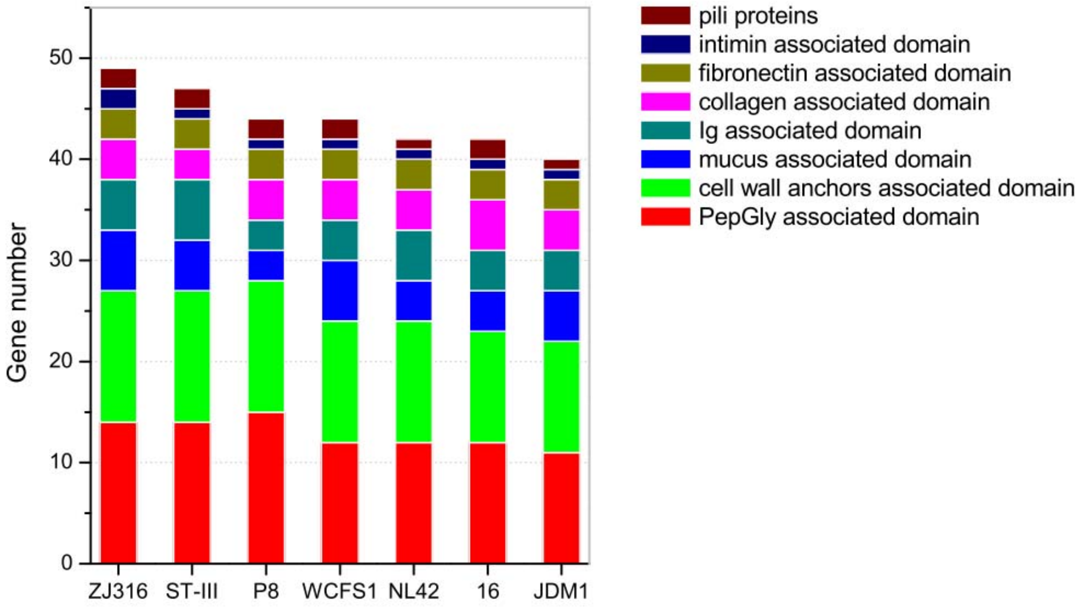

Fig. S4 Numbers of adhesion-associated and cell wall-anchored genes in different *Lt. plantarum* strains.

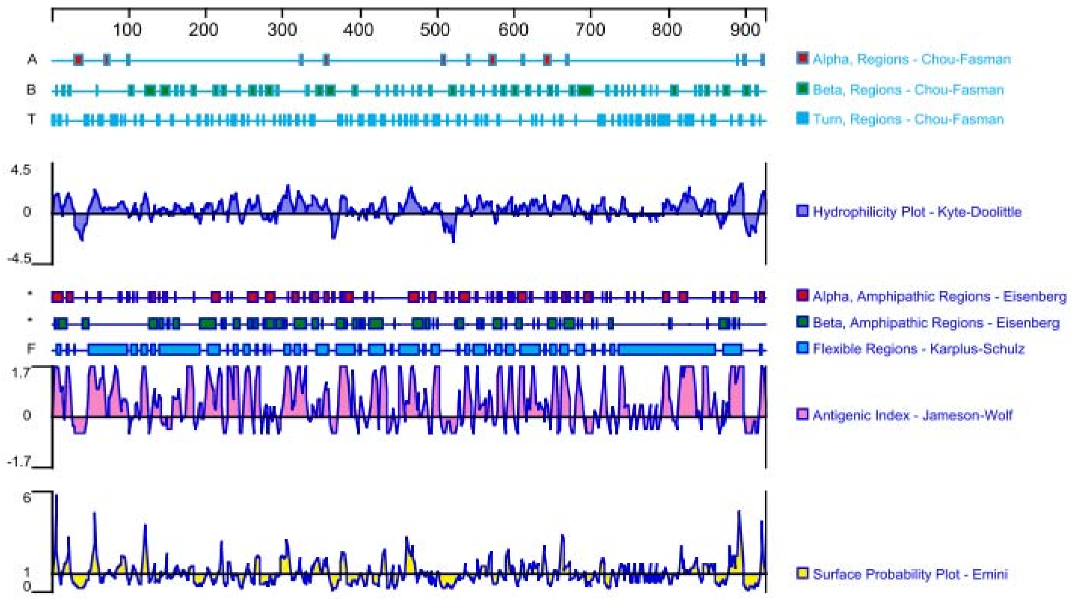

Fig. S5 Predicted secondary structure of CwaA. The analysis was performed using Protean (Lasergene package, DNASTAR, Madison, WI, USA).

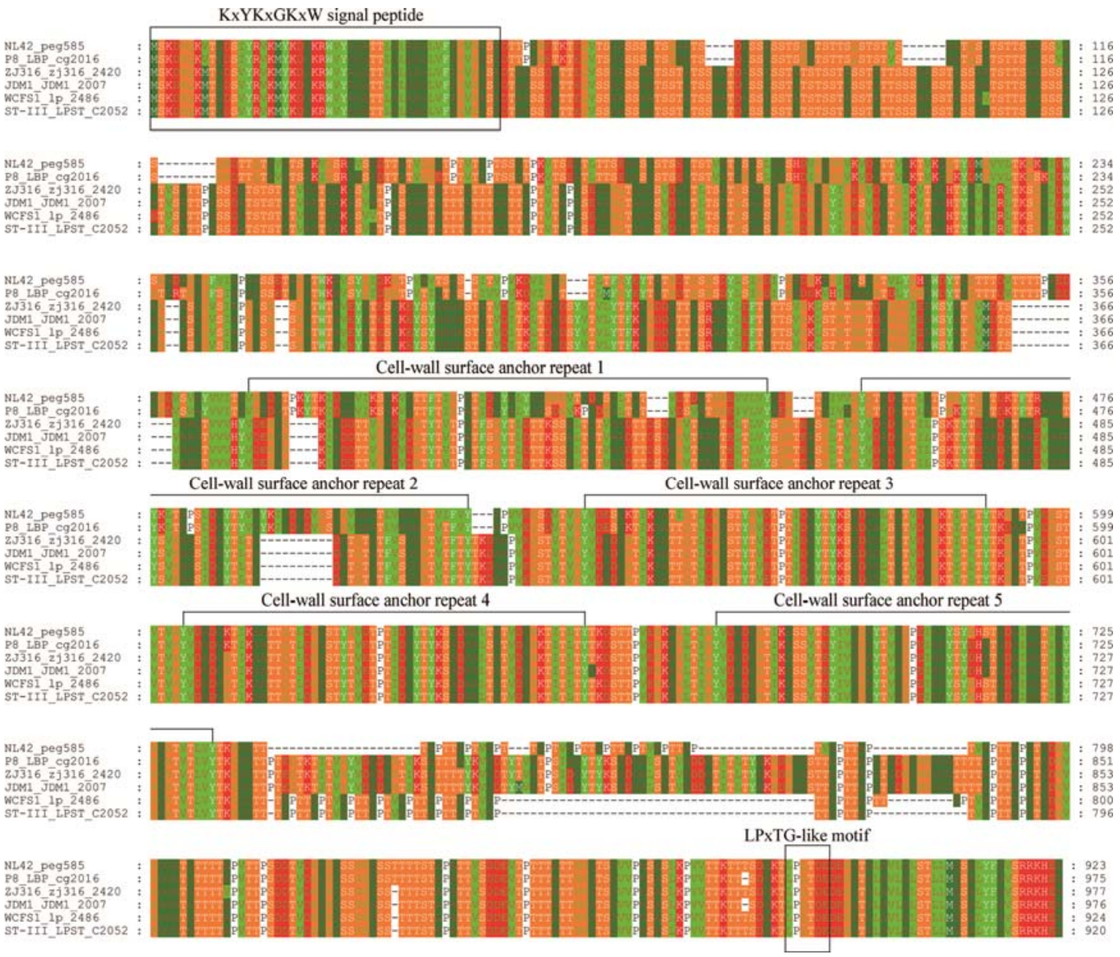

Fig. S6 Multiple sequence alignment of *Lt. plantarum* NL42 CwaA with its homologues in other *Lt. plantarum* strain complete genomes. Regions of signal peptide, cell wall surface anchor repeats and LPxTG-like motif are labeled. NL42_peg585 indicates CwaA.

1	11	21	31	41
MSKDNQKVTG	DSIYRVKMYK	DGKRWVYAGA	TTLALAAGLV	FANVNASADT
51	61	71	81	91
TPANETKTEQ	VTSGASSSAT	SQAATSDASS	ASSTSQATST	TSQSTSTVSA
101	111	121	131	141
ATQSATSTTS	AASSVASQND	TTQTAVATSA	KVQSRAVSAD	TTATVQQATP
151	161	171	181	191
TVTAPTSSAT	PKVTSEATLT	TSEAASGSTS	EATSTVATGS	GSLAASHDVN
201	211	221	231	241
ANNVKVDGTT	VAKTVKGQTY	DMQVVLTKNK	AIDWSNGDAN	GQFVIAPAGS
251	261	271	281	291
SETANGTWKA	VSYQIDKNTP	VAVTSGSSIT	VPVKDVINAN	TLTFNYEYTA
301	311	321	331	341
TNDTNSNSDY	LSINDIPNAD	NKANIDASGN	TVLYQHGWDY	TAQTTTDVTT
351	361	371	381	391
TTPAEDAQDV	SVAYVVITGV	NADGTPKYTK	IDGGVIKSGK	VGTTFTINPA
401	411	421	431	441
TIDGYALYGA	NNNVTGDASG	LTGTVDTDAT	NNEVVLVYAE	NTGIVVNYQT
451	461	471	481	491
ADGTTLATPN	QYTQGTDKTF	TRAGGTYKLT	APSLDGYTYV	GYKIGDGDVQ
501	511	521	531	541
SGNVAAGTLV	AGANTVIFVY	APVVEQSDVT	VNYVDESGKT	IKAATIQTLD
551	561	571	581	591
NGSTYNVDTP	TIDGYTYKSA	DAALTGTVDG	NKTITLTYTK	NATPVEQSTV
601	611	621	631	641
TVNYVDADGK	TIKAATTQTL	DNGSTYNVDT	PTIDGYTYKS	ADAALTGTVD
651	661	671	681	691
GNKTITLTYT	KDSTTPVENK	ANLTINYVDA	DGNTIKASSV	TEYIVGQAYT
701	711	721	731	741
VGQPEIAGYS	YNHSTGDAIA	GTIGYNGNTV	TLVYTKNGGT	TTAPTTAPTV
751	761	771	781	791
APTTAPTVAP	TTAPTTAPTV	APTTAPTVAP	TTAPTVAPTT	APGTGDNVNG
801	811	821	831	841
GGTGTTTTAP	VTTPSDDTVD	NGNGSSNNGS	STTTTSTAPA	TTVSDDEVTP
851	861	871	881	891
TTTATTNNGT	SGVVPASASL	KPVVTTKTTT	SDAKTLPQTD	EDENGTALAV
901	911	921		
LGLSTLLMGS	ALYFGVSRRK	HEA		

Legend:

The conservation scale:

1 2 3 4 5 6 7 8 9

Variable Average Conserved

X **- Insufficient data - the calculation for this site was performed on less than 10% of the sequences.**

Fig. S7 Analysis of the evolutionary conservation of amino acids in CwaA using the web server ConSurf (http://consurf.tau.ac.il/).

Table S1 Bacterial strains and plasmids used in this study

Strains/plasmids	Relevant characteristics	Source or reference
Lt. plantarum NL42	Isolate from cheese	Zuo et al. 2014
Lc. lactis NZ9000	MG1363 *pepN*::nisRK	Kuipers et al. 1998
Lc. lactis NZ9000-401	*Lc. lactis* NZ9000 harboring empty vector pNZ401	This study
Lc. lactis NZ9000-401-*cwaA*	*Lc. lactis* NZ9000 harboring pNZ401-*cwaA*	This study
E. coli DH5α	Cloning host	Takara
E. coli DH5α-401	*E. coli* DH5α harboring empty vector pNZ401	Laboratory stock
E. coli DH5α-401-*cwaA*	*E. coli* DH5α harboring pNZ401-*cwaA*	This study
E. coli H10407 (ETEC) ATCC 35401	Enterotoxigenic *E. coli* strain	ATCC[1]
S. aureus ATCC 25923	Quality control strain	CGMCC
pNZ401	Em^r, inducible expression vector containing the nisA promoter, derivative of pNZ8048	Laboratory stock
pNZ401-cwaA	pNZ401 carrying *cwaA* gene	This study

[1]http://www.atcc.org/products/all/35401.aspx
